# Cost-benefit analysis of feeding market dairy cows prior to slaughter compared with shipping direct

**DOI:** 10.3168/jdsc.2026-1041

**Published:** 2026-06-04

**Authors:** M.W. Overton, H.M. Goetz, S.M. Roche, J. Byrne, T.F. Duffield

**Affiliations:** 1 Zoetis Animal Health, Parsippany, NJ 07054; 2 ACER Consulting Ltd., Guelph, ON, Canada N1G 5L3; 3 Department of Population Medicine, University of Guelph, ON, Canada N1G 2W1; 4 Ontario Ministry of Agriculture, Food and Agri-business, Guelph, ON, Canada N1G 4Y2

## Abstract

Cull (market) dairy cattle often arrive at auction facilities and slaughterhouses in poor body condition, which presents welfare concerns and results in low return on carcasses at slaughter. The objective of this study was to evaluate the potential economic value of feeding market-designated dairy cows for 60 d before shipping compared with shipping directly to slaughter and determine sensitivity of this value to BCS, market price, and feed costs. A deterministic partial budget was created to estimate the costs associated with feeding market cows for 60 d, considering inputs related to initial and final BCS, management costs, and discounts applied for cull cattle at various BCS. Compared with a market cow shipped directly at a BCS of 2.0, a cow fed for 60 d to achieve a BCS of 3.0 would sell for approximately Can$664 greater. However, this benefit was partially offset by the associated costs of retaining her on the farm. Sensitivity analyses showed the greatest benefit-to-cost ratio (BCR) in feeding cows from a relatively low initial BCS when the cost of lactating cow TMR was low. Specifically, the highest BCR of 1.9 was seen when cows began with a BCS of 2.0 and the average cost of lactating cow TMR was Can$0.23/kg or Can$0.24/kg. A BCR of 1.7 was observed in cows with an initial BCS of 2.0 at market values of Can$4.00 or Can$4.50/kg. This economic model and associated sensitivity analyses show that it may be profitable, while also improving cow welfare, to dry off and feed market dairy cattle before shipping in certain circumstances, yet advisors and producers should consider the individual farm's capacity to retain market cattle beyond a typical culling management protocol.

## Highlights

• Feeding thin market dairy cattle for 60 days can increase sale value.
• Improving BCS may benefit both welfare and sale value.
• Economic gains depend on feed cost and market price.
• Farm capacity and management should guide decisions.

## Graphical Abstract

**Figure:**
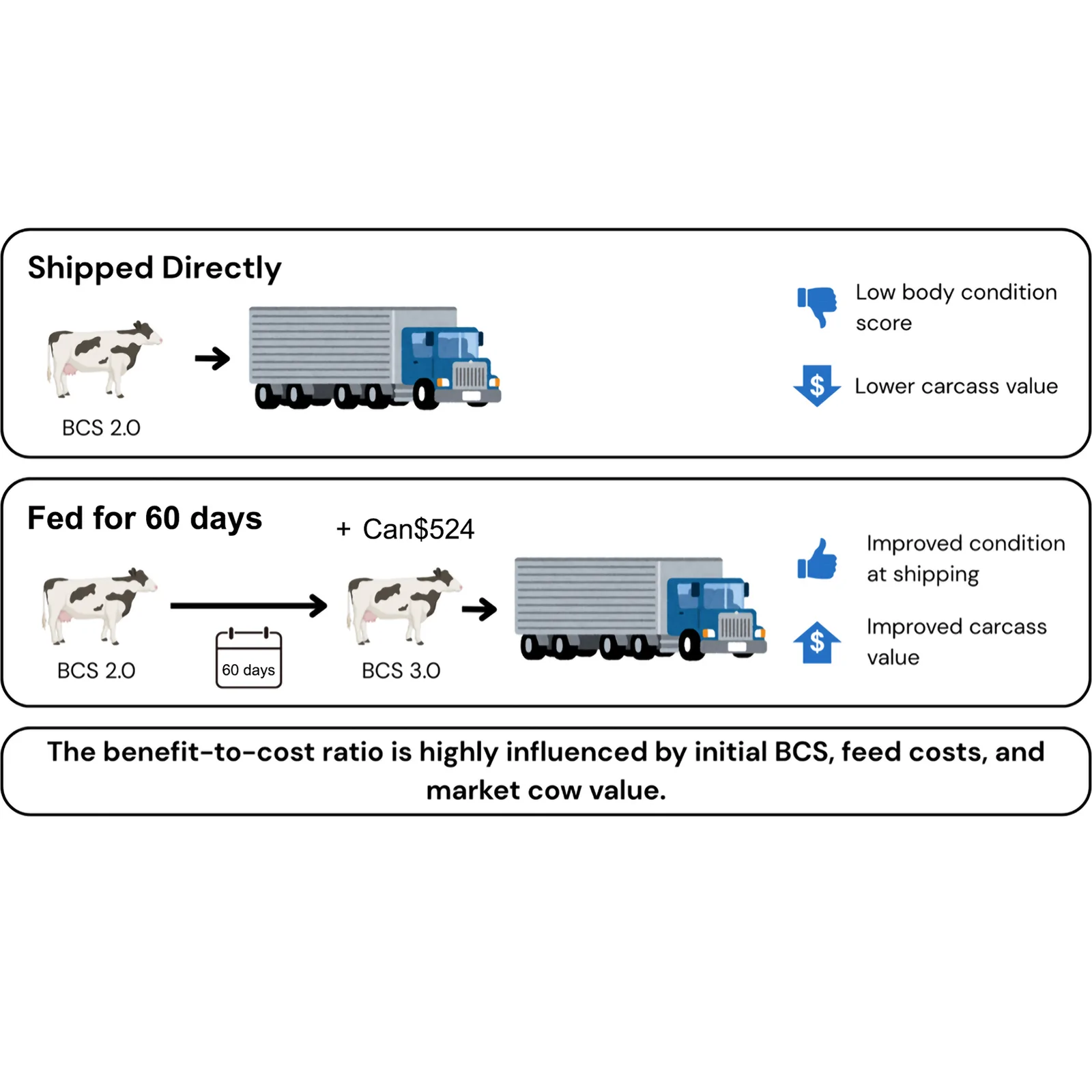
**Summary:** Cull (market) dairy cows are often shipped for sale or slaughter at a low body condition score (BCS), which can raise animal welfare concerns and reduce their market value. This study examined whether retaining these cows for 60 days to improve BCS before shipping could be economically worthwhile compared with shipping them directly. An economic model was built to compare the added income from improved BCS with the costs of feed and management during the feeding period. In this model, feeding a cow with a BCS of 2.0 to reach a higher BCS of 3.0 increased sale value. The highest benefit-to-cost ratio was seen in cows with a low initial BCS and when feed prices are relatively low or market prices are favorable. Overall, results suggest that feeding market dairy cattle before shipping may improve both animal welfare and farm profitability under the right conditions. Created with BioRender.com and Canva.com.

Approximately 30% to 40% of dairy cows are replaced annually in Canada due to decreased reproductive performance, low milk production, or health challenges (Lactanet, 2024). The marketing process of these animals often includes multiple transportation events after leaving the dairy farm of origin, with cull (market) cows frequently being sold through livestock markets before the final leg of transportation to an abattoir. Stojkov et al. (2020a) demonstrated that over half of the dairy market cows from British Columbia, Canada, were in the marketing system for more than 3 d, during which time their condition declined as they faced physiological strain resulting from comingling, exposure to novel environments, transportation, irregular access to food and water, and repeated loading and unloading. Further, cows were reported to be in the marketing system for as long as 16 d before slaughter with 2% of observed animals being sold on multiple days at separate auctions, highlighting the importance of ensuring fitness for transportation and marketing before shipping market dairy cattle (Stojkov et al., 2020b).

Body condition score and changes in BCS can be used to help approximate a cow's welfare state. Body condition score is correlated with an animal's energy reserves and is frequently assessed throughout the marketing process as an indicator of market cow condition (reviewed by Roche et al., 2009). Stojkov et al. (2020b) reported 10% of market dairy cows sold at 2 livestock markets in British Columbia as being very thin (BCS ≤2.0, on a 5-point scale), whereas Moorman et al. (2018) found 40.5% of culled dairy cows in 3 Ontario markets were very thin (BCS ≤2.0, on a 5-point scale), highlighting that the condition of Canadian market dairy cows varies but is generally less than optimal. Not only does arrival at auction facilities in poor condition present an animal welfare concern, but it also has significant economic impact: cows with low BCS risk downgraded carcass values and reduced liveweight sale prices. Specifically, in the aforementioned studies, market cows with BCS ≤2.0 sold for Can$0.63/kg and Can$0.20/kg less compared with cows with a BCS >2.0 (Moorman et al., 2018; Stojkov et al., 2020b), making BCS a key driver of economic return for producers and, potentially, demonstrating more ethical handling.

Recent changes to animal transportation regulations by the Canadian Food Inspection Agency in 2022 stipulate that cows with udder engorgement and lameness are compromised animals and therefore not fit for transportation as this would otherwise subject them to avoidable suffering (Government of Canada, 2022). These regulatory changes highlight efforts to improve animal welfare throughout the marketing process, as market dairy cattle often arrive to auction or slaughter in a compromised state: most commonly, studies report a high prevalence of low BCS, lameness, udder edema or engorgement, and other signs of illness or stress (Moorman et al., 2018; Stojkov et al., 2020b). Another opportunity for improving welfare of market dairy cattle is through implementing a nonlactating feeding period before shipment, which has been demonstrated to increase BCS and reduce udder engorgement scores (Berdusco et al., 2024a). Beyond the benefit of improving animal welfare, this strategy increases characteristics related to enhanced carcass quality and therefore the potential for higher economic returns for producers compared with shipping directly to slaughter (Berdusco et al., 2024b). Previous work has shown that feeding market cows for 60 d before shipping increased fitness for transport, carcass traits, and meat quality compared with cows sent directly to slaughter (Berdusco et al., 2024a,b). In this work, BCS improvement ranged from 0.5 to 2.0 points with an average of 1.2 BCS points gained during the feeding period (Berdusco et al., 2024a,b). Thus, strategic feeding of market cows before shipping could enhance body condition and market price, but data on the economic return of this practice are limited.

The objective of this study was to explore the economic value of feeding market dairy cows to improve BCS by at least 1 BCS point in a 60-d period before shipping compared with shipping directly to slaughter. The secondary objective was to determine the scenarios in which this practice would be most economically viable through cost-benefit sensitivity analyses. We hypothesized that feeding market dairy cows for 60 d would improve economic return compared with shipping directly to slaughter and that the benefit-to-cost ratio (**BCR**) would be highest for cows with a lower initial BCS.

Data regarding the impact of feeding market dairy cattle before shipping versus shipping directly to slaughter were collected by Berdusco et al. (2024a,b). In brief, market cows from a single dairy farm between February 2022 to February 2023 were randomly allocated into 2 treatment groups: cows shipped directly to slaughter or cows fed for 60 d before shipping. Dairy cows identified for replacement and randomized into the group to be fed were housed in a freestall pen with a pasture mat base covered in chopped straw bedding. Cows producing less than 22 kg/d at dry-off were administered teat sealants and dry cow antibiotics in all 4 quarters, then placed directly into the market cow pen. If the cow was producing more than 22 kg/d at dry-off, she was moved into a tiestall and milked 1×/d for 1 wk, then teat sealants and dry cow antibiotics were administered in all 4 quarters before being transferred to the market cow pen. Fed cows received a dry cow diet, consisting of straw, corn silage, haylage, and a dry cow supplement, for 2 wk and then a ration similar to a lactating cow TMR (consisting of straw, corn silage, haylage, corn, and a supplement) until slaughter. Feed samples were obtained 3×/wk from the dry and lactating cow diets to determine the average DMI on a weekly basis. Live weight and BCS were recorded at enrolment into the trial, as well as the day before transportation to the abattoir (located 20 km from the farm). Hock lesions (Berdusco et al., 2024a), udder engorgement (Stojkov et al., 2020a), locomotion (Berdusco et al., 2024a), and BCS (Vasseur et al., 2013) were scored weekly in fed cows and the day before slaughter in the fed and directly shipped cows.

A deterministic model of the cost of retaining a cull dairy cow for 60 d on a Canadian dairy farm before shipping was developed using Microsoft Excel (Redmond, WA; Supplemental File S1, see Notes). Model inputs were selected from the available literature where feasible or from personal communications with experts in this field and are outlined in Table 1. It was assumed that there is space within the current infrastructure of a dairy farm to house these cows without affecting other animals on the farm and that cows are on average second lactation animals when replaced. The Beef Farmers of Ontario Weekly Market Summary was used to identify the current dairy market cow price per 100 lb BW basis for the week of October 3, 2025, to October 9, 2025, and converted to Canadian dollars (Can$)/kg of BW for the model (Can$ to $US February 20, 2026 = 0.73). An initial BCS of 2.0 was selected as Moorman et al. (2018) reported a high frequency of market dairy cattle at Ontario auction markets with a BCS of ≤2.0 on a 5-point scale in 0.25 point increments, with the goal of representing the cost of increasing by at least 1 point of BCS by feeding for 60 d to achieve a BCS of 3.0 or greater. With an ADG of 1.95 kg/d (Berdusco et al., 2024a), the final BW achieved by this feeding strategy was estimated to be 817 kg. Berdusco et al. (2024a) reported a mean DMI of 21 kg/d when cows were fed the finishing diet for 53 d. Approximate cost of a typical lactating cow TMR was used from current nutrition industry contacts and discounted by 25% assuming that TMR refusals or orts would primarily be used for feeding market cows providing that sufficient quantities remain (Can$0.31/kg original TMR and Can$0.23/kg for refusals). In practice, producers might have lower or higher feed costs depending on several factors including feed availability, regional feed prices, and management practices. To estimate the value of a cow being shipped with a BCS of 2.0 without additional feeding to achieve a BCS of 3.0, a blended average discount was derived from Moorman et al. (2018), Ahola et al. (2011), and Stojkov et al. (2020b) to represent price discounts for cows with a BCS of 1.0 to 2.75 relative to a baseline BCS of 3.0. A corresponding premium of Can$0.06/kg for cows with a BCS of 3.25 to 4.0 was also derived from these sources. These discounts and premiums are described in Table 1 and reflect market cow discounts for cows with a BCS of 1.0 to 2.0 as compared with a BCS of 3.0, which reflects the average market cow value. Using these same sources, a premium was estimated of Can$0.06/kg for BCS of 3.25 to 4.0. There was no premium assumed for BCS >4.0. The estimated benefit and cost of this strategy when comparing a cow with BCS of 2.0 to a cow with BCS of 3.0 is outlined in Table 2. Specifically, feed costs were estimated by multiplying the estimated DMI by the cost of the associated diet to determine the cost of feeding the drying diet (dry cow TMR) for 14 d and feeding the finishing diet (good quality lactating cow TMR refusals) for 46 d. Yardage was estimated based on S. Hendrick (2025, Telus Agriculture, Lethbridge, Alberta. Canada, personal communication) to account for non-feed costs of maintaining cattle on a per-head, per-day basis. Disease risk was calculated based on multiplying the average treatment cost by the risk of mastitis reported in Berdusco et al. (2024a) and the cost of mastitis prevention (dry cow therapy with teat sealant) was Can$30. The losses associated with mortality were estimated by summing the lost original value of the cow being directly shipped to slaughter and the daily feed and yardage costs incurred by the cows that died up until the median days to death. The mortality risk was taken from Berdusco et al. (2024a).

**Table 1:** Input parameters and associated references and assumptions used to estimate the financial viability of feeding cull cows for 60 d before shipping on an Ontario dairy farm in Canadian dollars (Can$)

Variable name	Estimate	References and assumptions
Current market cow price^1^ (Can$/kg)	3.64	Beef Farmers of Ontario Weekly Market Summary
Initial BCS	2	
Initial BW (kg)	700	
Final BCS	3	
BW gain (kg)	117	1.95 kg/d (Berdusco et al., 2024a)
Final BW (kg)	817	
Estimate dry-off cost (Can$)	30	Assuming that cull cows are already dried off
Yardage/d (Can$/d)	0.75	Personal communication
Days on drying diet (d)	14	Assumed 7 d to fully dry off
Feed cost per kg DM (drying) (Can$/kg)	0.16	
Average DMI (drying) (kg/d)	15	
Days on finishing diet (d)	46	
Cost of lactating TMR (Can$/kg)	0.31	
Feed cost per kg DM (finishing) (Can$/kg)	0.23	Assuming a 25% discount of lactating TMR (refusals)
Average DMI (finishing) (kg/d)	21	Berdusco et al., 2024a
Disease risk (%)	8.7	Berdusco et al., 2024a
Average treatment cost (Can$)	31.38	Average cost of Spectramast^2^ LC (ceftiofur intramammary suspension) treatment protocol with once daily administration
Mortality risk (%)	4.3	Berdusco et al., 2024a
Median days to death (d)	14	

1 Baseline market cow price was set for market value for a cow with a BCS of 3.0 on a 5-point scale in 0.25 point increments (Vasseur et al., 2013). This was either discounted or increased based on this baseline as follows: BCS = 1.0 to 2.0 (−Can$0.34/kg); BCS = 2.25 to 2.75 (−Can$0.01/kg); BCS = 3.0 (referent); BCS = 3.25 to 4.0 (+Can$0.06/kg); BCS = 4.25 to 5.0 (Can$0.00).

2 Spectramast, Zoetis Animal Health, New Jersey.

**Table 2:** Estimated benefits and costs of 2 market cow management scenarios: (1) shipping directly to slaughter with a BCS of 2, and (2) feeding from an initial BCS of 2 to reach a BCS of 3 over a 60-d feeding period

Benefit	Shipped direct	Fed for 60 d	Difference
Slaughter weight (kg)	700	817	117
BCS (BCS points)	2	3	1
Price (Can$/kg)	3.30	3.64	0.34
Cow value (Can$)	2,310	2,974	664
Cost (Can$)			
Feed		(263.09)	
Yardage		(43.04)	
Disease risk		(2.73)	
Mastitis prevention		(30.00)	
Losses due to mortality		(103.00)	
Total costs			(442.00)

To assess how manipulation of the input parameters (initial BCS, average cost of lactating cow TMR, and average market cow value) affect the economic model's benefit and BCR and associated viability of feeding market dairy cows, sensitivity analyses were performed and described in Figure 1. The estimated BCR depending on lactating cow TMR and initial BCS ranged from 1.0 to 1.9, with the highest BCR in cows with an initial BCS of 2.0 and an average cost of lactating cow TMR of Can$0.23/kg or Can$0.24/kg. When considering the BCR depending on initial BCS and average market cow value, the estimated BCR ranged from 0.6 to 1.7. The highest BCR was identified in cows with an initial BCS of 2.0 and average market cow value of Can$4.00 or Can$4.50/kg.

**Figure 1:**
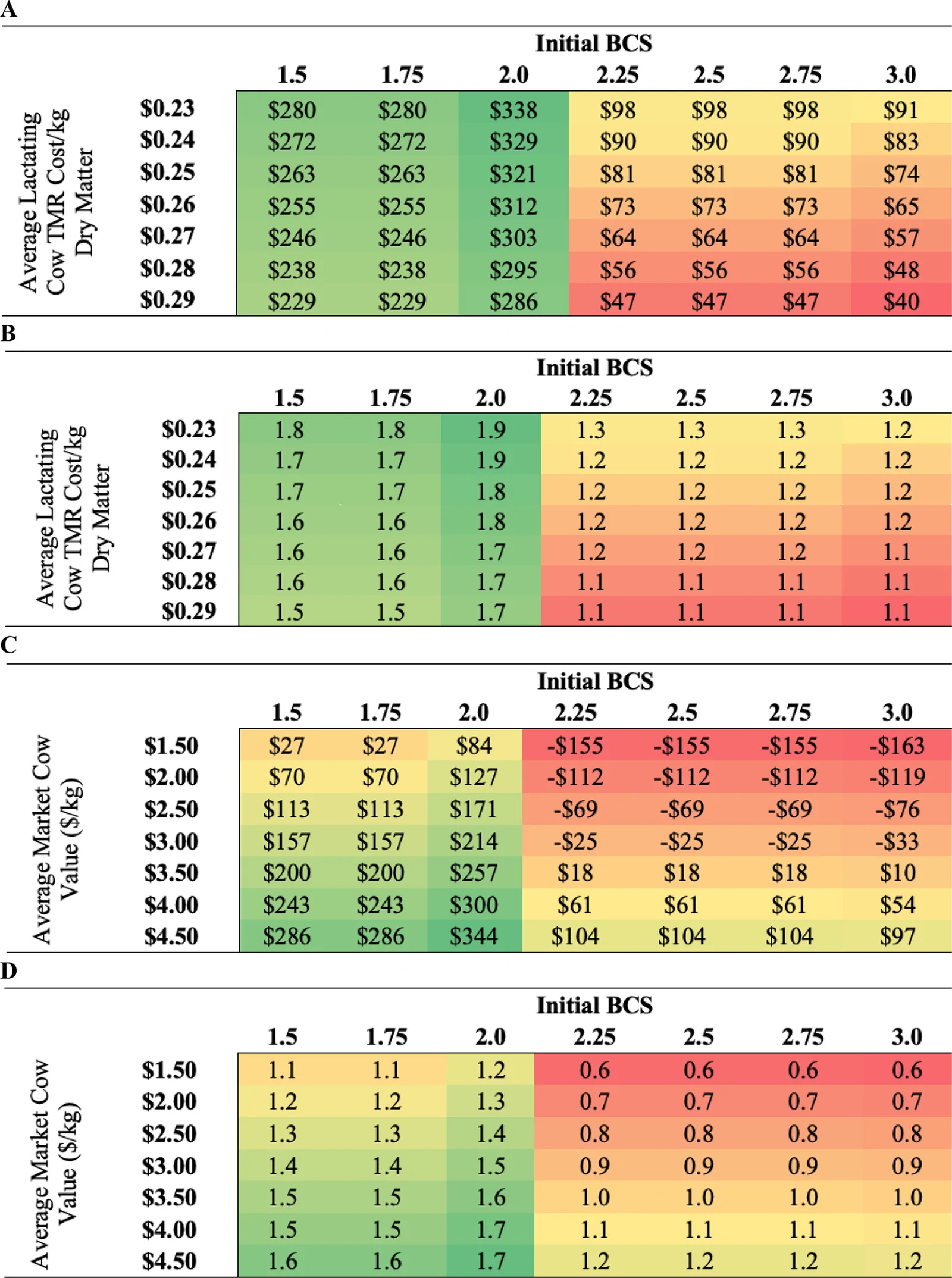
Sensitivity analyses demonstrating (A) the estimated benefit of feeding a cull dairy cow to increase 1 BCS depending on initial BCS and average cost of lactating cow TMR (Can$/kg of DM), (B) the benefit-to-cost ratio (BCR) of feeding a cull dairy cow to increase 1 BCS depending on initial BCS and average cost of lactating cow TMR, (C) the estimated benefit of feeding a cull dairy cow to increase 1 BCS depending on initial BCS and market price (Can$/kg) of market cattle, and (D) the BCR of feeding a cull dairy cow to increase 1 BCS depending on initial BCS and market price of market dairy cattle. All values in figure are Can$.

The proposed model in this study evaluated the economic implications of retaining cull dairy cows for a 60-d feeding period before marketing compared with shipping them directly to slaughter. Our findings indicate that this strategy may offer economic benefits under certain conditions; however, the feasibility and profitability should be carefully assessed within the context of each farm's resources, management, and market conditions rather than adopted universally.

The present economic model showed a potential benefit of feeding market dairy cattle to improve condition by 1 BCS point, with the greatest potential economic value being seen for feeding cull cows that begin with a low BCS (<2.25). Because there may be limited value in feeding better conditioned cows, this highlights the potential importance of earlier replacement decisions to capture optimal market value before BCS is lost in part due to delaying time to removal. On the other end of the BCS spectrum, cows with a BCS of 1.0 would not be candidates for shipping directly to slaughter or to auction, as the Dairy Cattle Code of Practice states that extremely thin animals (BCS <2.0 on a 5-point scale) are not fit for transportation (National Farm Animal Care Council, 2023). However, it is not uncommon to find market cows at auction between a BCS of 1.0 and 2.0 and these cows likely offer the greatest opportunity for weight gain from feeding before shipping. Lower economic returns are seen when aiming to increase BCS by 1 point in market cattle with an already reasonable BCS (>2.0), suggesting that this strategy may only be viable in specific situations, depending on other parameters such as feed costs and beef value of market dairy cows. Further, the economic benefit of feeding market dairy cattle before shipping is partly predicated on BW gain achieved per 1 point increase in BCS; however, there is inherent variability in weight gain per BCS between animals and can depend on lactation, stage of production, and other farm-specific dynamics (Maier et al., 2011; Lean et al., 2022). This variability introduces uncertainty into economic projections and can affect cost-effectiveness of feeding strategies before shipping.

Additionally, there are animal health parameters that play a key role in determining the suitability of a feeding period before shipping. Market dairy cattle are often replaced in the herd due to reduced production, lameness, or other limitations (Lactanet, 2024). Animals experiencing these health challenges may be less suitable for prolonged pre-shipping periods as this could compromise animal welfare. It is worth noting that loss from mortality accounts for 25% of the estimated cost for feeding these cows in our model. Carefully choosing healthy cows and providing proper health care can greatly minimize this risk and cost. Therefore, physical soundness must be considered alongside potential economic profitability when assessing whether a pre-shipment feeding strategy is viable.

This study was limited by the availability of data, specifically regarding the per kilogram discount for each respective BCS due to the scarcity of literature on the topic. Ahola et al. (2011) reported BCS and associated price in market dairy cows within a dataset of 23,479 animals sold in livestock markets in Idaho, California, and Utah. However, comparable data for market cows with each BCS sold in Canada are not available. Furthermore, previous research has demonstrated that market cow price is influenced by a range of factors beyond body condition. For example, Moreira et al. (2021) reported that price ratio was strongly associated with carcass merit traits and was also affected by factors such as lactation number, injury status, and production history of several dairy cattle sold at auction sites in Wisconsin. The absence of comparable, multifactor pricing data in the present study further limited the ability to fully account for variability in market value. An additional limitation of the model presented herein relates to the negative impact of retaining market cattle beyond a farm's typical replacement management strategy. When done appropriately, culling is the process of improving the herd by replacement, whereby cows leave the herd due to low milk production, infertility, or other reasons, and are replaced by more efficient animals (Overton and Dhuyvetter, 2020). This model does not capture the individual dairy herd capacity for cows and the potential implications of increased stocking density. For example, overcrowding of dairy cattle reduces milk production and milk fat, tends to increase herd somatic cell count, and can decrease fertility (Bach et al., 2008; Krawczel and Grant, 2009; Schefers et al., 2010). The economic impact of these effects on the overall herd was not captured by this model, and therefore, this model may only be applicable to a herd with existing capacity for retaining cull cattle without affecting other animals on farm. Additionally, producers may ultimately market cattle based on achieving a target BCS, rather than feeding for a fixed time period to achieve an increase in BCS of 1 point as described in the present study. However, the standardized increase modeled here allows for comparison of economic returns across cows entering the feeding period at differing initial condition. Future work could explore the economic implications of variable feeding periods tailored to individual target BCS at shipping.

In conclusion, feeding market dairy cattle before shipping presents an opportunity to improve animal welfare outcomes as it increases fitness for transportation in an otherwise potentially compromised population of animals. The economic value of this practice is dependent on a multitude of factors, primarily the feed cost, variable market price of cull dairy cattle, and individual farm factors such as capacity to house these cattle without affecting other animals in the dairy herd.
